# Alterations in the homeostasis of phospholipids and cholesterol by antitumor alkylphospholipids

**DOI:** 10.1186/1476-511X-9-33

**Published:** 2010-03-25

**Authors:** José M Jiménez-López, Pablo Ríos-Marco, Carmen Marco, Josefa L Segovia, María P Carrasco

**Affiliations:** 1 Department of Biochemistry and Molecular Biology I, Faculty of Sciences, University of Granada, Av. Fuentenueva s/n, Granada 18001, Spain

## Abstract

The alkylphospholipid analog miltefosine (hexadecylphosphocholine) is a membrane-directed antitumoral and antileishmanial drug belonging to the alkylphosphocholines, a group of synthetic antiproliferative agents that are promising candidates in anticancer therapy. A variety of mechanisms have been suggested to explain the actions of these compounds, which can induce apoptosis and/or cell growth arrest. In this review, we focus on recent advances in our understanding of the actions of miltefosine and other alkylphospholipids on the human hepatoma HepG2 cell line, with a special emphasis on lipid metabolism. Results obtained in our laboratory indicate that miltefosine displays cytostatic activity and causes apoptosis in HepG2 cells. Likewise, treatment with miltefosine produces an interference with the biosynthesis of phosphatidylcholine via both CDP-choline and phosphatidylethanolamine methylation. With regard to sphingolipid metabolism, miltefosine hinders the formation of sphingomyelin, which promotes intracellular accumulation of ceramide. We have demonstrated for the first time that treatment with miltefosine strongly impedes the esterification of cholesterol and that this effect is accompanied by a considerable increase in the synthesis of cholesterol, which leads to higher levels of cholesterol in the cells. Indeed, miltefosine early impairs cholesterol transport from the plasma membrane to the endoplasmic reticulum, causing a deregulation of cholesterol homeostasis. Similar to miltefosine, other clinically-relevant synthetic alkylphospholipids such as edelfosine, erucylphosphocholine and perifosine show growth inhibitory effects on HepG2 cells. All the tested alkylphospholipids also inhibit the arrival of plasma-membrane cholesterol to the endoplasmic reticulum, which induces a significant cholesterogenic response in these cells, involving an increased gene expression and higher levels of several proteins related to the pathway of biosynthesis as well as the receptor-mediated uptake of cholesterol. Thus, membrane-targeted alkylphospholipids exhibit a common mechanism of action through disruption of cholesterol homeostasis. The accumulation of cholesterol within the cell and the reduction in phosphatidylcholine and sphingomyelin biosyntheses certainly alter the ratio of choline-bearing phospholipids to cholesterol, which is critical for the integrity and functionality of specific membrane microdomains such as lipid rafts. Alkylphospholipid-induced alterations in lipid homeostasis with probable disturbance of the native membrane structure could well affect signaling processes vital to cell survival and growth.

## Review

### Introduction - Alkylphospholipid analogs as membrane-directed drugs

Alkylphospholipid (APL) derivatives are novel cytostatic agents that, in contrast to most of the currently used chemotherapeutic drugs, do not target DNA or the cytoskeleton but act at the cell membrane [1]. There is increasing interest in the biological activity of these lipid analogs as they selectively inhibit the growth of transformed cells and could well complement existing DNA-directed anticancer chemotherapies. Miltefosine (hexadecylphosphocholine) belongs to the alkylphosphocholine (APC) group, which exert antitumor activity against a broad spectrum of established tumor cell lines and solid tumors [2-5]. The inhibition of tumor cell proliferation caused by these agents may be the result not only of direct cell damage but also because of the induction of apoptosis [6]. Initial clinical studies have shown promising results; for example, miltefosine may be used for the topical treatment of cutaneous metastases of mammary carcinomas [7]. Remarkably, miltefosine exhibits potent leishmanicidal activity as a consequence of its interference with the parasite's metabolic pathways [8]; thus, orally administered miltefosine has been reported to be efficacious against the visceral and cutaneous forms of leishmaniasis [9,10]. Miltefosine is also toxic in vitro to other protozoan parasites [11-13].

**Figure 1:**
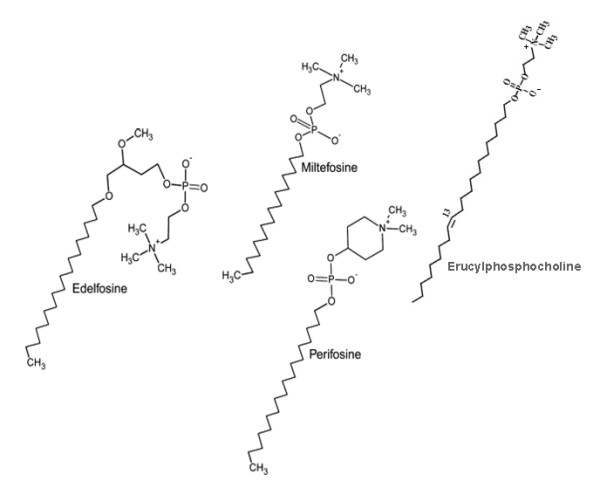
**Chemical structure of synthetic alkylphospholipids used in our studies**. The structures were adapted from [70].

Miltefosine is a representative member of a second generation of the synthetic APL family, being the prototype of the first generation edelfosine. In an attempt to improve antitumor activity with reduced side effects, erucylphosphocholine and perifosine appeared (Figure 1). Compared to miltefosine, erucylphosphocholine contains a longer hydrocarbon chain with a *cis *double bond and perifosine presents a piperidine moiety instead of the choline head group [1]. A wide variety of molecular mechanisms have been proposed to explain the antitumor activity of distinct membrane-directed APLs [14], whose action appears to depend on the cell type [15], the uptake rate into the cell [16] and the compound under study. Due to their chemical structure, APLs easily insert into lipid membranes and resist catabolic degradation; the level of partitioning into lipid bilayers depends on the degree of unsaturation of phospholipid alkyl chains and the amount of cholesterol. Miltefosine interacts with the cell membrane and rapidly reaches other subcellular membranes [17,18], being able to affect cell metabolism at different levels. The enzymes involved in lipid metabolism are mainly located in the membranes of the endoplasmic reticulum (ER) and thus would be a target for miltefosine activity. Until now its mode of action has not been precisely established, although the membrane appears to be the primary site of its activity, most likely due to interference with lipid metabolism and lipid-dependent signal transduction [1,8]. Effects induced by miltefosine upon a wide range of cellular processes such as the modulation of calcium homeostasis [19], alterations of phospholipase C [20], phospholipase A2 [21] or phospholipase D activity [22], lipid-signal transduction events [23,24] and phosphatidylcholine (PtdCho) metabolism (reviewed in [25]) has led to several hypotheses being put forward to explain how it works, one of these being that it may arrest tumor cell proliferation by interfering with the biosynthesis of PtdCho.

This review will mainly focus on the actions of miltefosine in the human hepatoma HepG2 cell line, a well-established model to examine hepatic lipid transport and metabolism, with emphasis on the alterations caused by miltefosine in the homeostasis of phospholipids and cholesterol. Additionally, the effects of other APLs with potential clinical relevance, such as edelfosine, erucylphosphocholine and perifosine will be also described.

### Effects of miltefosine on glycerolipid and sphingolipid metabolism

#### Influence of miltefosine upon glycerolipid metabolism

Preliminary studies in our laboratory demonstrated that nontoxic concentrations of miltefosine exert an antiproliferative effect on cultured HepG2 cells; e.g., a concentration of 50 μM miltefosine for 48 h caused a decrease in the number of cells, without a significant loss of viability, in the presence of serum. These findings agree with those encountered in MDCK [26], HeLa [27] and other neoplastic cell lines [2], indicating that HepG2 cells are moderately sensitive to the toxic effects (doses higher than 100 μM) and cytostatic activity of miltefosine concentrations in the micromolar range.

**Figure 2:**
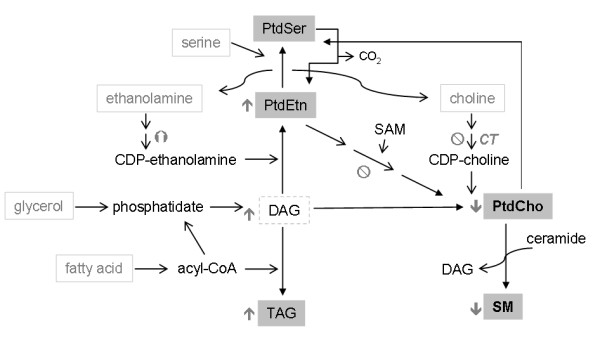
**Diagram for the hepatic biosynthetic pathways of phospholipids and triacylglycerol**. Several radiolabeled lipid precursors are depicted as outlined within the text. The postulated mode of action of miltefosine on specific metabolic steps is included. It is noteworthy that miltefosine inhibits the synthesis of both choline-bearing phospholipids, PtdCho and SM.

Our research group [28] and others [2,3,29] have shown that miltefosine inhibits the synthesis of PtdCho via CDP-choline (Figure 2). As far as the soluble intermediates in the CDP-choline pathway are concerned, we have found that treatment of HepG2 cells with miltefosine produces a significant increase in the label of choline phosphate and a decrease in that of CDP-choline compared to the untreated cells. Thus, the inhibitory effect produced by miltefosine on PtdCho synthesis in these cells seems to be the consequence of an alteration in CTP:phosphocholine cytidylyltransferase (CT) activity. In fact, the exposure of HepG2 cells to miltefosine caused a dose-dependent increase in cytosolic CT activity and this was accompanied by a concomitant decrease in membrane-bound CT activity in the cell particulate fraction, while the total CT activity was unaltered. Therefore, this APC interferes with PtdCho biosynthesis by impairing the translocation of the rate-limiting enzyme CT from the cytosol, where it is inactive, to membranes, where it expresses activity; that is, it affects only the distribution of CT. Miltefosine did not inhibit particulate CT activity in vitro, i.e., at the membrane level, but did inhibit cytosolic CT activity in the presence of low amounts of activating PtdCho/oleate liposomes. Thence, miltefosine appears to hinder the insertion of the soluble CT form into lipid vesicles or the membrane to become activated. Interestingly, simultaneous exposure of cells to oleate increased CT activity hereby stimulating PtdCho synthesis and it drastically reversed the inhibitory effect of miltefosine on PtdCho formation [28]. The reduction in PtdCho biosynthesis was shown not to be due to any alteration in choline uptake by the HepG2 cells, a finding which agrees with that found in MDCK cells [29], but it does go against observations made in neuronal cells [30] and KB and Raji cells [31]. In the latter cells, an increase in the degradation of PtdCho was also apparent after miltefosine treatment [31]. The inhibition of PtdCho synthesis in the HepG2 cell line after miltefosine incubation was not related to any alteration in the degradation rate of PtdCho or its secretion into the culture medium; in addition, treatment with miltefosine altered neither the activity of cytosolic choline kinase nor that of membrane-bound diacylglycerol cholinephosphotransferase [28].

It is noticeable that induction of apoptosis by distinct APLs in lymphoma cells occurs through inhibition of CTP:phosphocholine CT after internalization via raft-mediated endocytosis [32,33]. Since PtdCho is involved in cell-signaling processes, minor alterations in its levels may contribute negatively to cell survival. In fact, slight changes in PtdCho levels occurred in MDCK cells after 24 h of miltefosine treatment [34]. Having in mind that specific alterations in lipid metabolism could be involved in programmed cell-death processes, we have provided experimental evidence for the induction of apoptosis by miltefosine in HepG2 cells. After prolonged treatment with miltefosine, the tumoral cells showed a clearly rounded morphology and became increasingly detached from the plate. In addition, cells exposed to miltefosine for more than 24 h showed typical features of apoptosis, such as DNA laddering and caspase-3 activation. Thus, apoptotic cell death induced by miltefosine in the HepG2 cells appears to involve, at least, an increased caspase-3-like protease activity and genomic DNA fragmentation [28].

Incubation of HepG2 cells with miltefosine was also shown to increase the de novo biosynthesis of triacylglycerol (TAG) and PtdEtn [35]. The combined enhancement of TAG and PtdEtn syntheses may be attributed in part to the higher availability of diacylglycerol for glycerolipid biosynthesis when the synthesis of PtdCho is inhibited, as illustrates in Figure 2. On the other hand, the release of PtdCho into the medium remained unaltered while that of TAG increased somewhat in the presence of miltefosine, indicating that the apparent effects of miltefosine on cellular glycerolipid synthesis in the HepG2 cells were not due to an alteration of lipid secretion into the medium; in addition, miltefosine did not significantly affect metabolic turnover of prelabeled glycerolipids, e.g., PtdCho, as indicated above.

Concerning the synthesis of PtdEtn, we analyzed the water-soluble intermediates and final product, PtdEtn, of the CDP-ethanolamine pathway and found that miltefosine causes a modest increase in the incorporation of radiolabeled ethanolamine into CDP-ethanolamine and PtdEtn and a decrease in ethanolamine phosphate, which might be interpreted in terms of a stimulation of CTP:phosphoethanolamine CT activity, the rate-limiting enzyme of this metabolic pathway. Even though these changes might be attributed to miltefosine stimulating the synthesis of PtdEtn in HepG2 cells, the effect was quite slight, the radioactivity in PtdEtn increasing by only 10% compared to controls [36]. It has been reported that miltefosine treatment enhances the amount of PtdEtn in the membranes of *Leishmania donovani *promastigotes [10]. Moreover, the ether lipid edelfosine increases the production of CDP-ethanolamine and hence enhances PtdEtn synthesis in MCF-7 cells [37]. Since PtdEtn can be methylated in the ER to give PtdCho, we analyzed this process and observed that miltefosine significantly decreases the microsomal synthesis of PtdCho from PtdEtn by inhibiting PtdEtn *N*-methyltransferase activity [36]. These results constituted the first experimental evidence that the inhibition of the synthesis of PtdCho via CDP-choline by miltefosine is not counterbalanced by any increase in its formation via methylation. On the contrary, in the presence of miltefosine both pathways seem to contribute jointly to a decrease in the overall synthesis of PtdCho in HepG2 cells, as shown in Figure 2.

The uptake of radioactive serine into phosphatidylserine (PtdSer) and other phospholipids remained unchanged by miltefosine and neither was the activity of either PtdSer synthase or mitochondrial PtdSer decarboxylase (to give PtdEtn) altered, demonstrating that the biosynthesis of PtdSer is unaffected by miltefosine in HepG2 cells [36]. Treatment of the human lymphoma Raji cell line with miltefosine also led to an inhibition of PtdCho synthesis via CDP-choline, but enhanced, however, the generation of PtdCho from PtdSer via decarboxylation and methylation processes as a compensatory mechanism [38], suggesting that the effect of miltefosine on cellular phospholipid metabolism may well differ depending upon the cell type.

### Influence of miltefosine upon sphingolipid metabolism

With regard to sphingolipid metabolism, we found that exposure of HepG2 cells to miltefosine produces a marked time-dependent inhibition of sphingomyelin (SM) synthesis, using radiolabeled palmitate as exogenous substrate [39]. An accumulation of ceramide was observable after short-term miltefosine treatment, which could well be a result of diminished SM synthesis [35]. Thus, early treatment with the APC brings about a clear decrease in the SM/ceramide ratio. These results agree with findings reported in HaCaT cells, showing that the incorporation of choline into SM is inhibited by miltefosine concomitantly with an increase in intracellular ceramide levels [3]. Due to the precursor-product relationship, the biosynthesis of SM catalyzed by SM synthase might be influenced by the inhibition of the PtdCho synthetic pathway in the presence of miltefosine. On the other hand, incubation of HepG2 cells with miltefosine did not seem to alter the degradation of SM via sphingomyelinase activity, in accordance with previous observations in human leukemia cells [40].

Keeping a strict balance in the composition and relative proportions of phospholipids is of vital importance to the integrity of the cell membrane. Hence, it is worth emphasizing that early miltefosine treatment may affect lipid homeostasis and hereby cell membrane function by decreasing the synthesis of choline-bearing phospholipids, that is, PtdCho and SM (Figure 2), which are key membrane lipid components. Thus, subtle changes in these phospholipid pools may exert significant effects on the biological response of cells to miltefosine treatment.

### Disruption of cellular cholesterol transport and metabolism by alkylphospholipids

#### Overall picture of intracellular cholesterol trafficking and metabolism

Cholesterol is an essential constituent in the membrane of the mammalian cells, therefore abnormalities affecting cholesterol homeostasis result in several pathological conditions, notably atherosclerosis, Alzheimer's and Niemann-Pick type C (NPC) diseases. The cells obtain cholesterol by taking it up from their environment, mostly in the form of low-density lipoproteins or by de novo synthesis [41]. Cholesterol homeostasis in the hepatic cell is regulated by a complex set of mechanisms that include cholesterol biosynthesis, hydrolysis of cholesteryl esters (CE) from lipoproteins internalized into lysosomes, and transport of released cholesterol to intracellular organelles, such as the ER, for its esterification and conversion into bile acids [42]. Many aspects of the hepatic metabolism of cholesterol are well known, including its synthesis in the ER, its extracellular transport in plasma lipoproteins, its uptake by the low-density lipoprotein receptor (LDLR), and its regulation via sterol regulatory element-binding proteins (SREBPs). The pathways involved in cholesterol metabolism are strictly related to its transport and intracellular distribution among subcellular organelles and the plasma membrane (PM) [41]. Nevertheless, these pathways and their molecular regulation are still only partially understood. The difficulty comes from the interconnection between the different pools of intracellular cholesterol and the variety of mechanisms that can operate simultaneously to transport cholesterol within cells, including vesicular and nonvesicular transport [43].

**Figure 3:**
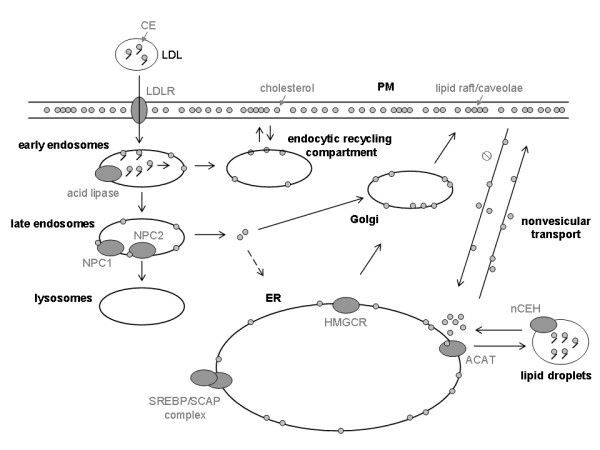
**A model for intracellular cholesterol trafficking in mammalian cells**. This figure was adapted from [42].

Three organelles are involved in cholesterol trafficking (Figure 3): (1) ER, the major site of synthesis, regulation and esterification of cholesterol, (2) PM, a prominent storage site for unesterified cholesterol, and (3) endosomes/lysosomes, where lipoprotein-derived cholesterol is liberated. Endocytosed LDL are delivered rapidly to lysosomes; the protein/phospholipid coat is degraded and CE are hydrolyzed to cholesterol [44]. Most, perhaps all, of this cholesterol is transported directly to the PM [45], which contains approximately 65-80% of the unesterified cholesterol in the cell [46]. Cholesterol synthesized in the ER, as well as that released in the endosomes/lysosomes by lipoprotein catabolism, moves to the PM against a steep concentration gradient [41,47]. Once the capacity of the PM and other compartments to absorb cholesterol is exceeded, cholesterol is transported back to the ER, where it is esterified by acyl-CoA:cholesterol acyltransferase (ACAT), regulates 3-hydroxy-3-methylglutaryl-CoA reductase (HMGCR) proteolysis, and inhibits proteolytic processing of SREBP2, which is required for expression of sterol-regulated genes [48]. This distribution of cholesterol between sites of regulation, synthesis, and deposition provides an efficient control for cellular cholesterol levels. With the exception of the SCAP (SREBP cleavage-activating protein) system, the complex control mechanisms for regulating cholesterol levels in different cellular compartments remain largely unknown.

Maintenance of the cycle between free and esterified cholesterol relies on the bidirectional transport of sterols between the ER and the PM and/or an endocytic compartment. Proper control of this transport is important for normal cell function and development, as indicated by fatal human pathologies such as NPC disease and atherosclerosis, which are characterized by an over-accumulation of free sterols within the endosomal membranes and the ER, respectively [49]. Two independent NPC genes responsible for this neurodegenerative disorder have been identified, but the precise functions of the encoded NPC1 and NPC2 proteins remain unknown [50]. It is clear now that cholesterol travels from the PM to the ER via a different route from that taken by the nascent cholesterol leaving it [41,51]. Plasma-membrane cholesterol is thought to follow at least two pathways to the ER: (1) a vesicular route via endosomes and (2) a nonvesicular alternative route. Some amphiphilic drugs, such as U18666A, progesterone and imipramine, have been described as interfering with intracellular sterol traffic by accumulating lysosomal unesterified cholesterol in fibroblasts and Chinese Hamster ovary cells [52,53]; the movement of cholesterol from the cell surface to the ER is inhibited by these drugs, which also affect cholesterol transport from the endosomes [54]. This defect in cholesterol transport causes cell damage and mimics the cellular lesions observed in fibroblasts from patients affected by NPC disease, characterized by the accumulation of unesterified cholesterol and other lipids in the endosomal/lysosomal compartment [55-57].

#### Effects of alkylphospholipids on cellular cholesterol transport and metabolism

**Figure 4:**
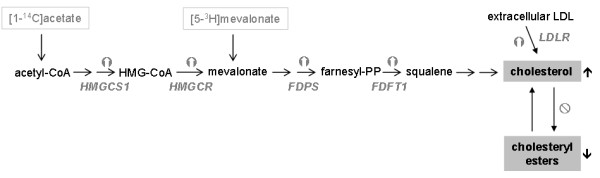
**Basic scheme for cholesterol biosynthesis from radiolabeled acetate or mevalonate as precursors**. The postulated mode of action of miltefosine on specific metabolic steps is included. Enzyme abbreviations: HMGCS1 (cytosolic HMG-CoA synthase); HMGCR (HMG-CoA reductase); FDPS (farnesyl diphosphate synthase); FDFT1 (farnesyl-diphosphate farnesyltransferase 1, also known as squalene synthase); LDLR (low-density lipoprotein receptor).

Our group has extensively examined the effects of the antitumoral drug miltefosine on intracellular cholesterol transport and metabolism and its relevance in maintaining cholesterol homeostasis. It was shown that treatment of HepG2 and Vero cells with miltefosine (also erucylphosphocholine) significantly alters cholesterol metabolism and leads to an accumulation of cholesterol in the cell [35]. In fact, free cholesterol levels increased up to 3-fold when HepG2 cells were exposed to miltefosine for 48 h. Using radiolabeled substrates we determined the effect of this APC on cholesterol synthesis, the destiny of cholesterol from LDL and the transport of cholesterol between the PM and the ER. Long-term exposure of HepG2 cells to miltefosine caused a marked increase in cholesterol biosynthesis when acetate, but not mevalonate, was used as the lipogenic precursor, thereupon emerging HMGCR as a prime target for miltefosine (Figure 4). This enzyme is an integral protein of ER membranes, which is rate-limiting for cholesterol synthesis and catalizes the production of mevalonate from HMG-CoA. HMGCR is mainly regulated at the transcriptional level through a negative-feedback mechanism by the ER cholesterol pool [48]. Remarkably, treatment with miltefosine induced higher activities of HMGCR and the cell-surface LDLR in HepG2 cells, in both a concentration- and time-dependent manner. Furthermore, the steady-state protein levels and mRNA expression of HMGCR and LDLR increased after incubation with the APC. HMGCR activity was not, however, directly modulated by miltefosine as assayed in vitro both in HepG2 cell lysates and rat-liver microsomes, and exposure of cells to miltefosine did not significantly affect the decay rate of the reductase [58]. On the other hand, incubation with miltefosine did not significantly change the expression of ACAT. These data indicate that miltefosine stimulates the cholesterogenic pathway as well as the receptor-mediated uptake of cholesterol in HepG2 cells via an increase in the expression of HMGCR and LDLR, respectively.

Uptake of cholesteryl linoleate in LDL and further hydrolysis of these esters increased following exposure of HepG2 cells to miltefosine. However, cholesterol esterification from several radioactive exogenous fatty acids was markedly reduced after treatment with miltefosine, even in the presence of the cholesterol analog 25-hydroxycholesterol added to the culture medium; in addition, the reesterification rate of cholesterol from LDL decreased after treatment with the APC [35,58]. This inhibition of intracellular CE synthesis was observable after only one hour of exposure to miltefosine [39]. In general, the concentration of CE is a result of the balance between cholesterol esterification and hydrolysis of CE. The latter mainly results from the activity of neutral CE hydrolase (nCEH), which resides in cytosolic lipid droplets, while the ER-localized enzyme ACAT is responsible for the esterification of excess cholesterol, and hence plays a key role in cellular cholesterol homeostasis (Figure 3). We investigated the action of miltefosine on the synthesis and hydrolysis of CE in cell homogenates and found that ACAT activity appears to decrease in response to miltefosine, since a reduction of fatty-acid incorporation into CE was observed [35]. Afterwards, we confirmed that the apparent inhibition of ACAT after treatment with miltefosine was due to a depletion of ER-associated cholesterol pool [58]. On the other hand, nCEH activity was not affected when the cells were exposed to miltefosine [35].

All these observations point to the fact that miltefosine in general alters cholesterol metabolism. The observed increase in cholesterol synthesis brought about by miltefosine treatment and the decrease in cholesterol esterification disturb the intracellular cholesterol/CE cycle and lead to high levels of unesterified cholesterol in HepG2 cells. An increased content of cholesterol in membranes from miltefosine-treated *Leishmania donovani *promastigotes has also been reported [10], nevertheless it involves exogenous cholesterol recruitment within the parasite membranes and appears to depend on a direct interaction between miltefosine and sterols [59].

It has been suggested that CE might serve as a dynamic reservoir, its synthesis being controlled by ACAT activity, which would be regulated by the supply of cholesterol in the ER [42]. Bearing in mind that cholesterol esterification is a measure of the levels of free cholesterol arriving at the ER, our data suggested that miltefosine treatment could interfere with CE synthesis owing to a reduction in the amount of plasma-membrane cholesterol fluxing to the ER and hereby lower cholesterol levels in the ER regulatory pool. Thus, we used the degree of esterification of plasma-membrane cholesterol as a marker of retrograde cholesterol movement from the cell surface to the ER, the site of ACAT activity [42,60]. Treatment with miltefosine was found to produce a marked decrease in the esterification of plasma-membrane cholesterol, indicating that the movement of cholesterol from the PM to the ER is disrupted by miltefosine [58]. Particularly, we showed that esterification of plasma-membrane cholesterol in cells exposed to miltefosine for a short 1-h period of time was also acutely reduced, and this effect was similar both in control and energy-depleted cells, hereby demonstrating that the APC alters mainly the nonvesicular intracellular cholesterol transport [39].

In addition to synthesizing cholesterol, mammalian cells also synthesize substantial amounts of precursor sterols. Similar to cholesterol, the precursor sterols leave the ER and rapidly reach the PM [61,62] and then move back to the ER to be enzymatically processed to cholesterol, being this movement essential to complete cholesterol biosynthesis. It has been shown that when traffic of cholesterol from the PM to the ER is disrupted, the circuit for synthesis of this sterol is affected, so intermediates of cholesterol biosynthesis are accumulated [62,63]. In fact, we found that the contribution of desmosterol and 7-dehydrocholesterol to the total C27 sterol synthesis increased 4-fold in HepG2 cells after 24 h of treatment with miltefosine, hereby increasing the ratio of sterol biosynthetic intermediates to cholesterol, the final product of this pathway [39]. Therefore, our results suggest that this APC not only interferes with the intracellular cholesterol traffic but also with the transport of these intermediates back to the ER for completion of cholesterol synthesis.

Hydrolysis of SM in the PM by treatment of HepG2 cells with exogenously added bacterial sphingomyelinase resulted in an enhanced fluxing and esterification of PM-associated cholesterol into the ER, as previously reported in other cell lines [64]; however, simultaneous exposure to sphingomyelinase did not prevent the inhibition of retrograde PM-to-ER cholesterol traffic and CE formation brought about by miltefosine treatment [39]. Moreover, the increase induced by miltefosine in the cholesterogenic activity and the inhibition of cholesterol esterification also occurred in the presence of exogenously added LDL-cholesterol [58]. On the contrary, exposure to this drug had no effect on the arrival of endosomal/lysosomal cholesterol to the PM, released from LDL internalized via the clathrin-mediated endocytic pathway, or the movement of endogenously synthesized cholesterol from the ER to the cell surface, where it can be extracted using cyclodextrin as aceptor. In order to confirm that the results were not a unique or unusual effect specific to HepG2 cells, we also made experiments with Vero cells and found a similar profile of changes induced by miltefosine upon the synthesis of cholesterol and CE [39]. Thus, the main mechanism by which miltefosine impairs cholesterol homeostasis and causes the accumulation of cholesterol within the cell is altering the nonvesicular, energy-independent transport of cholesterol from the PM to the ER (Figure 3).

Our most recent observations indicate that exposure to nonlytic concentrations of other membrane-active APLs such as edelfosine, erucylphosphocholine and perifosine decrease the proliferation rate of cultured HepG2 cells. Likewise, these agents alter intracellular cholesterol transport and metabolism in a manner similar to miltefosine, that is, they impaire cholesterol trafficking from the PM to intracellular membranes and, as a result, produce a remarkable decrease in cholesterol esterification, hence leading to an enhancement of cholesterol synthesis and LDL-cholesterol uptake in the hepatoma HepG2 cell line [65].

Cholesterogenesis is known to be transiently induced by the translocation of the transcription factor SREBP2 from the ER membrane (125-kDa precursor form) to the nucleus (70-kDa mature form) [66]. In fact, incubation of cells with miltefosine or edelfosine stimulated gene expression of SREBP2 as well as transcription of the SREBP2-responsive LDLR gene. In addition, exposure to miltefosine or edelfosine increased the mRNA transcript levels of cholesterol-synthesizing enzymes such as cytosolic HMG-CoA synthase (HMGCS1), HMGCR, farnesyl diphosphate synthase (FDPS) and farnesyl diphosphate farnesyltransferase-1 (FDFT1, also known as squalene synthase) (Figure 4), which are transcriptionally-regulated, rate-limiting enzymes of the cholesterol biosynthetic pathway [67]. Moreover, incubation of HepG2 cells with the different APLs produced a time-dependent increase of mature SREBP2 form in the assayed cell lysates as well as increased protein levels of its targets HMGCR and LDLR. As a likely consequence, cell exposure to miltefosine enhanced the content of cholesterol mainly in the membrane raft fractions (isolated by a detergent-free disruption procedure) and hence the cholesterol/SM ratio was clearly increased by miltefosine treatment, as compared to the untreated cells [65].

Cholesterol and SM are major lipid constituents of membrane raft microdomains, and the ratio cholesterol/SM is crucial to maintain the integrity of lipid rafts and thence membrane functionality. Consequently, the disturbance of this ratio could alter several signaling pathways associated to these membrane domains [68,69] and be involved in the biological actions exhibited by miltefosine and other APLs in different cell types.

## Conclusions

The bulk of our data indicates that miltefosine impairs cholesterol arrival into the ER, without altering reverse cholesterol trafficking from the ER to the PM, leading to a depletion of free cholesterol in the ER and consequently a deregulation of cholesterol biosynthesis and receptor-mediated cholesterol uptake. The final result of this interference is an increased uptake, synthesis and accumulation of cholesterol within the cell. Together with the reduction in PtdCho and SM syntheses induced by miltefosine, all these effects lead to an alteration in the choline-containing phospholipid/cholesterol ratio that can disturb membrane stability and function, and thus might be expected to inhibit tumor cell growth. Therefore, the imbalance in this ratio may well be partly responsible for the induction of apoptosis and the antiproliferative activity exhibited by this APC in HepG2 cells. We have recently found that other membrane-directed APLs such as edelfosine, erucylphosphocholine and perifosine also alter intracellular cholesterol homeostasis. Cholesterogenic response induced by APLs in HepG2 cells involves an increased gene expression and higher levels of several proteins related to the pathway of biosynthesis as well as the receptor-mediated uptake of cholesterol. All these alterations may affect membrane lipid composition and their distribution in raft-nonraft domains.

## Abbreviations

ACAT: acyl-CoA:cholesterol acyltransferase; APC: alkylphosphocholine; APL: alkylphospholipid; CE: cholesteryl esters; CT: cytidylyltransferase; ER: endoplasmic reticulum; HMGCR: 3-hydroxy-3-methylglutaryl-CoA reductase; LDLR: low density lipoprotein receptor; nCEH: neutral cholesteryl ester hydrolase; PM: plasma membrane; PtdCho: phosphatidylcholine; PtdEtn: phosphatidylethanolamine; PtdSer: phosphatidylserine; SM: sphingomyelin; SREBP: sterol regulatory element-binding protein; TAG: triacylglycerol.

## Competing interests

The authors declare that they have no competing interests.

## Authors' contributions

All authors participated in the design of these studies and carried out the different assays. JMJL drafted the manuscript. All authors read and approved the final manuscript.
